# HARMFUL ALGAL BLOOMS: Musty Warnings of Toxicity

**DOI:** 10.1289/ehp.118-a473

**Published:** 2010-11

**Authors:** Kris S. Freeman

**Affiliations:** **Kris S. Freeman** has written for *Encarta* encyclopedia, NIH, ABCNews.com, and the National Park Service. Her research on the credibility of online health information appeared in the June 2009 *IEEE Transactions on Professional Communication*

On hot August days it’s not uncommon for Midwestern swimmers and boaters to find their favorite freshwater beaches covered with musty scums of cyanobacteria, photosynthetic microbes formerly known as blue-green algae. Sometimes cyanobacteria produce toxins, an event called a harmful algal bloom (HAB). A recent survey of cyanobacterial blooms in 23 Midwestern lakes by the U.S. Geological Survey (USGS) suggests the odoriferous compounds that often accompany HABs may serve as sentinels of risk in recreational waters.^1^

Cyanobacteria produce a complex mixture of hepatotoxins and neurotoxins. Some species also produce relatively nontoxic “taste-and-odor” compounds such as geosmin and 2-methylisoborneol (MIB). These compounds, also found in soil and mushrooms, have strong earthy tastes and odors that people can detect at water concentrations of 10 ppt or less.^3^

In their preliminary study, Jennifer L. Graham and colleagues found geosmin and/or MIB co-occurred with cyanotoxins in about 91% of the blooms tested.^1^ Because cyanotoxins occurred more frequently than geosmin and MIB, the authors cautioned that taste and odor can’t provide surefire warnings of toxicity. However, a USGS press release on the study highlighted the need for increased cyanotoxin surveillance during taste-and-odor events so the public can be advised if necessary.^4^

The possibility of using taste-and-odor cues to help detect the presence of cyanotoxins is good news, because visual cues are notoriously equivocal. “A nasty-looking bloom doesn’t necessarily mean a toxic bloom,” says Mary Skopec, stream monitoring coordinator for the Iowa Department of Natural Resources. On the other hand, toxins may be left in the water after a bloom blows offshore or disperses, even if the water looks safe.

In the United States, recreation is the primary route of cyanotoxin exposure. Between 2005 and 2009 at least 19 states issued health advisories or closed recreational areas due to HABs, which can look like floating pools of blue, green, red, or brown paint.^5^,^6^ Recreational exposure can cause gastroenteritis, rashes, asthmalike symptoms, abnormal liver function, weakness, and dizziness.^2^ In countries where water treatment may be unreliable or unavailable, contaminated drinking water has caused serious disease and death in humans—for instance, exposure through drinking water has been linked to liver cancer in China.^2^

Children are at greater risk of recreational exposure than adults because “they tend to spend more time in the water and swallow more water,” says NIEHS toxicologist Michelle J. Hooth. Livestock and pets also are more vulnerable, attracted by the same earthy smell that repels humans.^7^ “Dogs can suffer seizures and die within minutes of coming out of [contaminated] water,” Graham says. “It’s very traumatic for a dog owner. People call us and want to know why they didn’t know about the danger.”

Angela Shambaugh, an aquatic biologist for the Vermont Department of Environmental Conservation, says concerns over two pet deaths in 1999 provided impetus for the initiation of cyanotoxin testing at Lake Champlain (which straddles New York, Vermont, and Quebec) and a successful communications program, including weekly online updates.^8^ This summer, when “we had an expansive and very colorful cyanobacteria bloom on the main portion of the lake, most residents, though unfortunately not all, knew to keep children and pets out of the algae,” Shambaugh says.

The U.S. Environmental Protection Agency has not set standards for cyanotoxin exposure for either recreational or drinking water, although it has added microcystin-LR and other cyanotoxins to its drinking water Contaminant Candidate List for further research.^9^ “For most cyanotoxins we don’t have enough toxicological data to come up with good guidelines,” Graham says. Moreover, the environmental factors that trigger any given bloom to produce high levels of toxin are complex and difficult to predict.^2^

Cyanotoxins can be effectively removed from drinking water by a variety of treatment procedures.^2^ But treatment is not foolproof, and in August 2010 microcystin-LR was detected in finished drinking water from three Ohio water systems, although none of the incidents were severe enough to warrant a drinking water advisory.^10^ “The issue of cyanotoxins is on the radar for everyone that uses surface water,” says Chris Jones, laboratory supervisor at Des Moines Water Works in Iowa, which draws drinking water from the Raccoon and Des Moines rivers.

The potential utility of taste and odor as a signal of toxicity is complicated by the fact that a musty smell doesn’t necessarily mean treated tap water is unsafe. Treatments that successfully remove cyanotoxins may not eliminate geosmin and MIB,^11^ which Jones says are “very water soluble.” And if a HAB develops more quickly than the Des Moines Water Works can ramp up treatment with activated carbon, he says, “We can get calls from people saying the water tastes like dirt.”^12^

But even though geosmin and MIB are not perfect indicators of presence of cyanotoxins, musty smells combined with the presence of cyanobacterial blooms can still serve as a “good warning tool” for recreational waters, says Keith Loftin, a coauthor of the USGS paper. “People can tell very quickly if something looks and smells bad.”

## Figures and Tables

**Figure:**
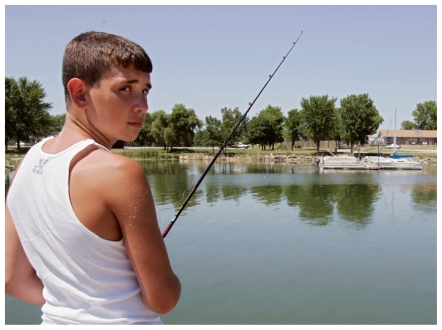
A boy fishes in Pawnee Lake near Lincoln, Nebraska, on 1 July 2005. HAB alerts were posted for Pawnee Lake for 14 weeks of the summer of 2005.
